# Generation of Reporter-Expressing New World Arenaviruses: A Systematic Comparison

**DOI:** 10.3390/v14071563

**Published:** 2022-07-18

**Authors:** Lucie Fénéant, Anne Leske, Karla Günther, Allison Groseth

**Affiliations:** Laboratory for Arenavirus Biology, Friedrich-Loeffler-Institut, Südufer 10, 17493 Greifswald-Insel Riems, Germany; luciestephanie.feneant@fli.de (L.F.); anne.leske@fli.de (A.L.); karla.guenther@fli.de (K.G.)

**Keywords:** reporter-expressing virus, reverse genetics, arenavirus, Tacaribe virus, virus detection, fluorescence, luminescence, imaging

## Abstract

Replication-competent reporter-expressing viruses are crucial tools in molecular virology with applications that range from antiviral screening to live-cell imaging of protein spatiotemporal dynamics. However, there is currently little information available regarding viable strategies to develop reporter-expressing arenaviruses. To address this, we used Tacaribe virus (TCRV), an apathogenic BSL2 arenavirus, to assess the feasibility of different reporter expression approaches. We first generated trisegmented TCRV viruses with either the glycoprotein (GP) or nucleoprotein (NP) replaced by a reporter (GFP, mCherry, or nanoluciferase). These viruses were all viable, but showed marked differences in brightness and attenuation. Next, we generated terminal fusions with each of the TCRV proteins (i.e., NP, GP, polymerase (L), matrix protein (Z)) either with or without a T2A self-cleavage site. We tested both the function of the reporter-fused proteins alone, and the viability of corresponding recombinant TCRVs. We successfully rescued viruses with both direct and cleavable reporter fusions at the C-terminus of Z, as well as cleavable N-terminal fusions with NP. These viruses all displayed detectable reporter activity, but were also moderately attenuated. Finally, reporter proteins were inserted into a flexible hinge region within L. These viruses were also viable and showed moderate attenuation; however, reporter expression was only detectable for the luminescent virus. These strategies provide an exciting range of new tools for research into the molecular biology of TCRV that can likely also be adapted to other arenaviruses.

## 1. Introduction

Arenaviruses that infect mammals (i.e., members of the genus *Mammarenavirus*) have bisegmented, single-stranded negative-sense RNA genomes and use an ambisense coding strategy to express their four viral proteins [1]. Their large segment (L-segment) encodes for the viral polymerase (L) and the matrix protein (Z), while their small segment (S-segment) encodes for the nucleocapsid protein (NP) and the glycoprotein precursor (GPC). During infection, the GP1 subunit of the mature glycoprotein interacts with the cellular receptor to mediate internalization [2,3], while the GP2 subunit mediates subsequent pH-dependent fusion in late endosomes, leading to the release of the viral genome into the cytoplasm [4,5]. Viral transcription and replication are then mediated by NP, which is responsible for RNA binding, and L, which synthesizes new RNA. Increasing expression of Z during the course of the infection operates as a switch from viral RNA synthesis to assembly through its interaction with L, which inhibits its polymerase activity [6]. Z then mediates the trafficking of encapsidated viral genomes and drives budding of new viral particles through interaction with ESCRT pathway components [7]. GPC matures in the endoplasmic reticulum (ER) and Golgi network through proteolytic cleavage into the GP1 and GP2 subunits, as well as a stable signal peptide (SSP), which together assemble into the mature glycoprotein complex for trafficking to the plasma membrane and incorporation into budding particles [8,9].

Despite this simple lifecycle and limited coding capacity, arenaviruses include a number of important human pathogens. In particular, the New World arenaviruses include a number of closely related agents responsible for causing severe hemorrhagic and neurological disease in humans with high case fatality rates in South America. To date, the most clinically significant of these has been Junín virus (JUNV), which is endemic in rural agricultural areas of Argentina and was responsible for up to 3000 cases per year before vaccination became available [10]. Even today, the disease remains endemic with a small number of cases reported on an annual basis. Further, increasing cases of the related Machupo virus in Bolivia during recent years [11], as well as the uncertain impact of political instability in Venezuela on Guanarito infections [12], both of which cause clinically similar diseases within their respective endemic areas, are a growing cause for concern. While protocols for the treatment of JUNV using neutralizing antibody-containing convalescent patient serum have been well-established, this approach has obvious practical limitations and, in particular, faces challenges due to the decreasing availability of donors as a result of the successful JUNV vaccination programs [13]. Worryingly, erratic funding to produce the vaccine also poses a continuous threat to the future success of such efforts [14]. Furthermore, similar resources for other related viruses remain unavailable.

These challenges clearly highlight the necessity to find new treatments against the diseases caused by these viruses [15]. At the same time, however, their extreme pathogenicity and associated classification as BSL4 agents makes antiviral discovery approaches using these viruses technically challenging and limits access to equipment and expertise needed for such approaches. While model systems have been developed in recent years to allow mid- to high-throughput screening efforts targeting these pathogens [16,17,18,19,20], follow-up validation in a viral context remains an important part of such efforts. One viable approach to facilitate screening efforts under BSL2 conditions, also on a larger scale, is the use of closely related surrogate viruses, such as the Tacaribe virus (TCRV), that exhibit little or no pathogenic potential in humans. Unfortunately, there are currently few resources available for such work and, in particular, traditional virus titration-based analysis of virus infection is cumbersome and presents a challenge to the use of these viruses in larger-scale applications. For other virus families, the development of reporter-protein-expressing viruses has proven valuable for antiviral drug discovery efforts [21,22,23,24,25,26,27,28], as well as downstream mechanism of action studies [29,30], and even the identification of host factors required for the viral lifecycle [31,32,33,34], which can themselves then be leveraged as targets for treatment. Further, for arenaviruses, recent work has shown that the development of reporter protein-expressing arenaviruses is possible [35,36,37,38,39,40,41,42,43,44], and some have even been successfully used for high-throughput screening [35,36,44]. However, to date, few approaches have been tested. 

Importantly, many features of the reporter virus design strategy affect its utility in different applications. In particular, the choice of reporter protein determines many technical aspects of the detection and defines the necessary experimental infrastructure. Further, both reporter intensity and expression kinetics are dependent on a number of factors, including not only the reporter used but also the expression strategy and location within the viral genome [38,40,41]. Further, the expression strategy affects reporter distribution, particularly in approaches focused on directly coupling reporter proteins to viral proteins, which can be valuable for investigating mechanism of action for some antiviral approaches, as well as for studying virus biology itself. Finally, these different approaches can also have markedly different impacts on virus attenuation, which is an especially critical issue for in vivo work. Consequently, these issues highlight the importance of comparing different strategies for the generation of reporter-expressing viruses to determine the best options for specific applications.

To accomplish this, we used a systematic approach to compare both different reporter genes (i.e., GFP, mCherry, and nanoluciferase (nLuc)) and different expression approaches. Here, we focused either on expression as a separate open reading frame (ORF) or expression together with each of the viral proteins, either as a directly-linked fusion protein or as a cleavable fusion using a 2A self-cleaving peptide. Based on this approach, we identified several new approaches for fusing reporters to the individual viral proteins, including some that are also viable in the context of recombinant TCRVs.

## 2. Materials and Methods

Cells: Vero76, HEK293T, and Huh7 cells were grown in Dulbecco’s Modified Eagle’s Medium (DMEM) supplemented with 10% fetal calf serum (FCS), 100 U/mL penicillin, and 100 μg/mL streptomycin (P/S; Thermo Fisher Scientific, Darmstadt, Germany). BSR-T7/5 cells were grown in Glasgow’s Minimal Essential Medium with Earle’s salts (GMEM) supplemented with 5% newborn calf serum (NCS) and P/S. Cells were maintained at 37 °C with 5% CO_2_. 

Plasmids: pCAGGS expression plasmids for TCRV NP, Z, GPC, NP-Flag, and GPC-Flag (strain TRVL-11573) have been previously described [45]. The pCAGGS expression plasmid for TCRV L was generated from viral RNA isolated using standard molecular biology approaches. These plasmids were then used for the further generation of expression constructs with the respective viral proteins either N- or C-terminally fused with GFP, mCherry, or nLuc reporters. These constructs were generated so that the reporter protein was either directly linked to the viral protein via a flexible linker sequence (GGGGGSG), or by a thosea asigna virus 2A (T2A) self-cleaving peptide (EGRGSLL/TCGDVEENPGP). Where T2A fusions were made to the viral protein C-terminus, a linker was also placed upstream of the T2A site (i.e., GGGGGSGEGRGSLL/TCGDVEENPGP). For the internal fusion of the reporter with TCRV L, a flexible hinge region—analogous to that described for other NSVs [46,47,48]—was identified within the polymerase based on its low sequence conservation among different arenaviruses (Supplementary Figure S1). Based on this alignment, an insertion site between amino acids 927/928 was selected and the reporters were inserted with Type IIS restriction enzymes using standard molecular biology methods.

For rescue of recombinant TCRVs, plasmids encoding the complete TCRV-L segment (Lseg) and TCRV-S segment (Sseg) in cRNA orientation were constructed based on amplification of the respective segments from isolated viral RNA and inserted into the previously published pAmp plasmid containing a T7 promoter upstream, and an HDV ribozyme followed by a T7 terminator element downstream of the insert [49] (i.e., pAmp-TCRV-Sseg; pAmp-TCRV-Lseg). To distinguish recombinant viruses from the natural isolate, silent mutations removing a BspMI restriction site in the L segment (C6882T; GenBank Accession MT081317) and a BamHI restriction site in the S segment (T2830C; GenBank Accession MT081316) were introduced. Protein ORFs with fused reporter proteins where cloned seamlessly into either pAmp-TCRV-Sseg or pAmp-TCRV-Lseg in place of the corresponding wild-type (wt) protein ORF for rescue of the corresponding reporter-expressing TCRVs. Alternatively, for the rescue of trisegmented reporter-expressing TCRV viruses, derivatives of pAmp-TCRV-Sseg were generated in which either the NP ORF or the GPC ORF was deleted and replaced by a small cloning locus containing Type IIS restriction sites. GFP, mCherry, or nLuc reporter genes were then inserted to seamlessly replace either the NP or GPC ORF, again using standard molecular biology methods. 

Monocistronic TCRV minigenome plasmids were generated based on pAmp-TCRV-Sseg but where both the NP and GPC ORFs were removed, as described above, and either GFP or nLuc was introduced into the GPC ORF. Similarly, for generating transcription and replication-competent virus-like particles (trVLPs), a bicistronic TCRV minigenome was used in which the TCRV NP ORF was replaced by GPC fused C-terminally via a T2A cleavage site to nLuc (GPC-T2A-nLuc), and the TCRV GPC ORF was replaced by TCRV Z. 

Virus rescue: Co-cultures of BSR-T7/5 and Vero76 cells (1:1) in 6-well plates were transfected at a confluence of ~60% with pAmp-TCRV-Sseg (1 µg), pAmp-TCRV-Lseg (1 µg), pCAGGS-TCRV L (1 µg), pCAGGS-TCRV NP (250 ng), and pCAGGS-T7 (250 ng), using Transit-LT1 (Mirus) with a ratio of 5 µL to 1 µg of DNA. After 24 h, the medium was replaced with GMEM + 2% NCS. Supernatants were harvested 7 days post-transfection and used to infect fresh Vero76 cells at a confluence of ~80% in 6-well plates. After incubation at 37 °C for 1 h, fresh DMEM + 2% FCS was added. Cells were incubated at 37 °C and observed daily for the development of cytopathic effects, at which point supernatants were harvested and used to infect new Vero76 cells in T150 flasks for production of working stocks. All recombinant viruses were fully sequenced to confirm that their sequences were correct.

Growth kinetics and reporter expression: Vero76 cells seeded for ~80% confluence in 24-well plates were infected with the indicated viruses at a multiplicity of infection (MOI) of 0.005 in serum-free DMEM for 1 h at 37 °C. Cells were then rinsed with serum-free DMEM and fresh DMEM + 10% FCS was added. Supernatants were harvested either immediately thereafter (day 0) or on the indicated days and titrated by plaque assay. Here, Vero76 cells seeded for a confluence of ~90% in 12-well plates were infected with serial dilutions of the indicated viruses in serum-free DMEM for 1 h at 37 °C. The inoculum was then removed and an overlay of MEM + 2% FCS and 0.7% agarose was added. Plaque assays were fixed in 10% formalin containing crystal violet ten days postinfection. For analysis of reporter expression, pictures were taken for fluorescent viruses on a Leica DMi8 fluorescence microscope (Leica Microsystems, Mannheim, Germany) using the same settings (i.e., exposure time, aperture, gain, etc.) for sets of images taken over several days. For luciferase-expressing viruses, cells were lysed in 1% Triton-X100 and luciferase activity was measured using Renilla-Glow Juice (PJK, Kleinblittersdorf, Germany) reagent in a Glomax Multi (Promega, Walldorf, Germany) microplate reader. 

Minigenome assay: BSR-T7/5 cells seeded for a confluency of ~60% were transfected in 12-well plates with either a nLuc or GFP-expressing monocistronic TCRV minigenome (125 ng), pCAGGS-T7 (125 ng), pCAGGS-TCRV L (500 ng), pCAGGS-TCRV NP (125 ng) and pCAGGS-firefly-luciferase (FLuc, 50 ng; as a transfection control) using Transit-LT1 with a ratio of 3 µL to 1 µg of DNA. For assays assessing the inhibitory activity of TCRV Z, 1 to 250 µg of pCAGGS-TCRV Z were additionally transfected. After 48 h, cells were lysed in 1% Triton-X100, and luciferase activity was measured using Renilla-Glow Juice (for nLuc) and Beetle Juice (PJK, for FLuc) reagents in a Glomax Multi microplate reader. For GFP-based minigenome assays, pictures of the entire well were taken using a Leica DMi8 fluorescence microscope and raw integrated densities for the GFP channel were measured using ImageJ.

trVLP assay: BSR-T7/5 cells seeded for a confluency of ~30% were transfected in 6-well plates with the bicistronic TCRV minigenome expressing GPC-T2A-nLuc and Z (250 ng), pCAGGS-T7 (250 ng), pCAGGS-TCRV L (1 µg), pCAGGS-TCRV NP (250 ng), and pCAGGS-FLuc (100 ng) using Transit-LT1 at a ratio of 3 µL to 1 µg of DNA. Supernatants were harvested after 72 h and used to infect Huh7 cells transfected the day before at a confluence of ~50% in 96-well plates with pCAGGS-TCRV NP (15 ng) and pCAGGS-TCRV L (55 ng). After 72 h, cells were lysed in 1% Triton-X100, and luciferase activity was measured using Nano-Glo (Promega) reagent in a Glomax Multi microplate reader.

Cell–cell fusion assay: BSR-T7/5 cells seeded for a confluence of ~60% in 24-well plates were transfected with pCAGGS-TCRV GPC (250 ng) and pCAGGS-mCherry (250 ng) or pCAGGS-GFP (250 ng). After 24 h, cells were treated for 5 min at 37 °C with HMS buffer (100 mM NaCl; 15 mM HEPES; 15 mM MES; 15 mM succinate; 2 mg/mL glucose) adjusted to the indicated pH. Cells were rinsed and fresh medium was added. After 24 h, cells were fixed in PBS + 2% paraformaldehyde (PFA) for 30 min at room temperature. Syncytia formation (≥3 nuclei) was measured using an Eclipse Ti-S fluorescence microscope equipped with NIS-Elements imaging software (version 4; Nikon, Düsseldorf, Germany). The total syncytia area was obtained by multiplying the number of syncytia by the mean syncytia area from 10 random fields of view.

Budding assay: HEK293T cells seeded for a confluence of ~50% in 6-well plates were transfected with pCAGGS-TCRV GPC-Flag (1000 ng), pCAGGS-TCRV NP-Flag (1000 ng), and pCAGGS-TCRV Z (or the indicated TCRV Z reporter construct; 1000 ng) using TransIt-LT1 with a ratio of 3 µL to 1 µg of DNA. Four days post-transfection, supernatants were harvested, cleared, and centrifuged at 280,000× *g* for 3 h on a TNE (10 mM Tris; 100 mM NaCl; 0.1 mM EDTA; pH 7.4)-20% sucrose cushion, and VLPs were resuspended in PBS. VLP producer cells were lysed in 1% Triton X-100. Samples were mixed with 4× SDS gel loading buffer (10% SDS (*w*/*v*); 40% glycerol (*v*/*v*); 20% β-mercaptoethanol; 0.008% Bromophenol Blue; 250 mM Tris-HCl, pH 6.8) and heated at 95 °C for 5 min. Budding efficiency was assessed by SDS-PAGE and semidry Western blot, using polyvinylidene difluoride membranes. Membranes were blocked for 1 h at room temperature (RT) in 10% skim milk diluted in Tris-buffered saline containing 0.1% Tween-20 (TBS-T). Primary antibodies (anti-TCRV NP; 1:10,000 [50] and anti-vinculin; 1:2000; sc-73614 from Santa Cruz (Heidelberg, Germany)) were incubated in 1% skim milk in TBS-T overnight at 4 °C. Secondary antibodies (anti-guinea-pig-HRP; 1:5000; DAB-087875 from Dianova (Hamburg, Germany), and anti-mouse-HRP; 1:5000; #7076 from Cell Signaling Technology (Danvers, MA, USA)) were incubated in 1% skim milk in TBS-T for 45 min at RT. Signals were detected using Clarity Western Substrate (Bio-Rad, Munich, Germany). 

Assessment of protein localization: For high-magnification pictures of intracellular protein localization, BSRT7/5 cells seeded on glass coverslips for a confluence of 30% in 12- or 24-well plates were transfected as described for minigenome assays or cell–cell fusion assays, respectively. The next day, cells were fixed for 30 min in PBS + 2% PFA and permeabilized for 10 min in PBS + 0.1% Triton X-100. Coverslips were mounted using DAPI-containing Prolong Diamond mounting solution (Invitrogen, Darmstadt, Germany) and images were taken with a Leica DMi8 fluorescence microscope.

## 3. Results

### 3.1. Trisegmented-Reporter Viruses

**Figure 1:**
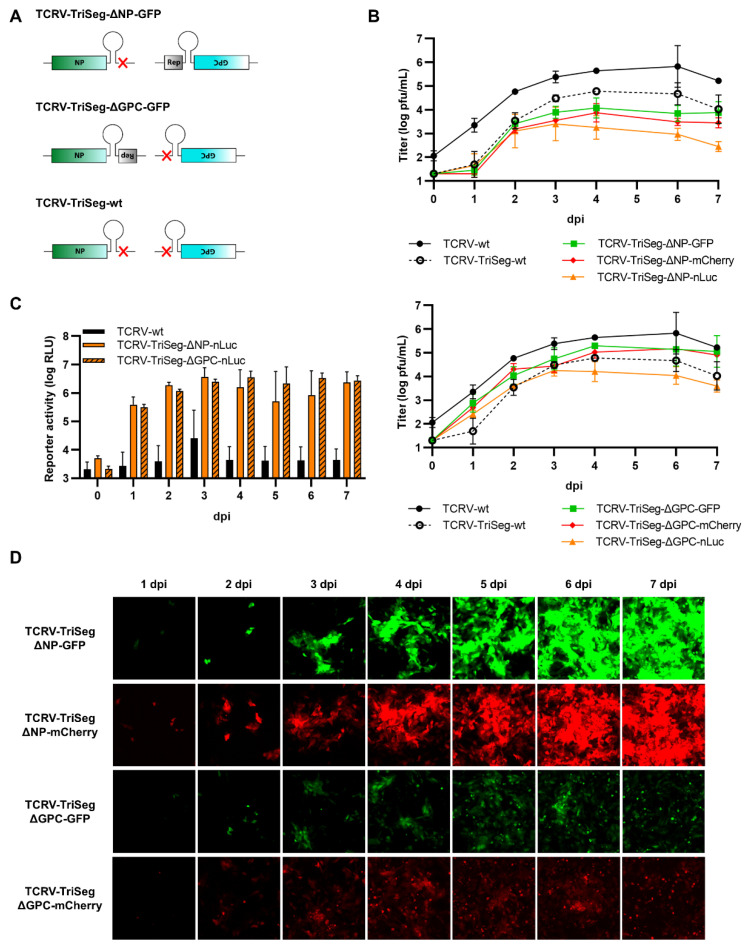
Reporter-expressing trisegmented Tacaribe virus (TCRV). (**A**) Schematic representation of trisegmented TCRV genomes. TCRV-TriSeg-∆NP-reporter viruses have a wild-type (wt) Lseg and two Sseg: one in which the NP ORF has been replaced by the reporter and one in which the GPC ORF has been deleted (top). TCRV-TriSeg-∆GPC-reporter viruses have a wt Lseg and two Sseg: one in which the GPC ORF has been replaced by the reporter, and one in which the NP ORF has been deleted (middle). The TCRV-TriSeg-wt virus has a wt Lseg and two Sseg: one in which the NP ORF has been deleted and one in which the GPC ORF has been deleted (bottom). (**B**) Growth kinetics. Vero76 cells were infected at MOI = 0.005 with TCRV-TriSeg-∆NP-reporter viruses (top) or TCRV-TriSeg-∆GPC-reporter viruses (bottom). Supernatants were harvested on the indicated days, and titrated by plaque assays. Results are shown as mean ± SD of two independent experiments. (**C**) Reporter activity (nLuc). Cells infected as in (**B**) were lysed in 1% Triton on the indicated days, and nLuc activity in lysates was measured. Results are shown as mean ± SD of three independent experiments. (**D**) Reporter activity (GFP/mCherry). Pictures of cells infected as in (**B**) were taken on the indicated days, at 10× magnification. Results are shown as representative images from one experiment out of two independent experiments.

Based on our recent sequencing data including corrected genome ends [51], we were able to establish a system for the rescue of recombinant TCRV, using a T7-driven approach analogous to that published previously by ourselves and others [49,52,53,54,55,56]. Further, consistent with previous publications showing that arenaviruses can incorporate more than two genomic segments during packaging, we could adapt our rescue system for production of reporter-expressing trisegmented TCRVs using an approach analogous to that used to generate trisegmented reporter-expressing Lymphocytic choriomeningitis virus (LCMV) [38] and, more recently, also TCRV [41]. In line with our goal to compare different reporters and expression strategies, we then generated viruses encoding versions of the Sseg with each of the three reporters—GFP, mCherry, and nLuc—replacing either the NP ORF (i.e., TCRV-TriSeg-∆NP-reporter viruses, Figure 1A, top) or the GPC ORF (i.e., TCRV-TriSeg-∆GPC-reporter viruses, Figure 1A, middle). The second copy of the Sseg encoded only the missing ORF. As a control, a trisegmented TCRV lacking any reporter genes (i.e., TCRV-TriSeg-wt, Figure 1A, bottom), as well as TCRV-wt, were also rescued. 

While all of the trisegmented TCRV viruses tested were viable, they also all showed impaired growth in Vero76 cells compared with TCRV-wt (Figure 1B). Interestingly, TCRV-TriSeg-∆NP-reporter viruses were consistently more impaired and reached lower titers than TCRV-TriSeg-∆GPC-reporter viruses. However, this was not further impacted by whether or not they expressed a reporter protein. Reporter expression from nLuc-expressing viruses was clearly visible already from 1 dpi and was fairly comparable between TCRV-TriSeg-∆NP and TCRV-TriSeg-∆GPC reporter viruses (Figure 1C). In contrast, we observed that reporter activity for fluorescent TCRV-TriSeg viruses, which became visible between 2–3 dpi, was clearly brighter for TCRV-TriSeg-∆NP reporter viruses than for TCRV-TriSeg-∆GPC reporter viruses (Figure 1D), despite their higher degree of attenuation. Thus, taken together, our data indicate that, while expression of different reporters from trisegmented TCRVs is a broadly viable strategy, both the choice of reporter and its position in the genome affect both reporter expression and the replication efficiency of the resulting virus.

### 3.2. Reporter Fusions with TCRV NP

**Figure 2:**
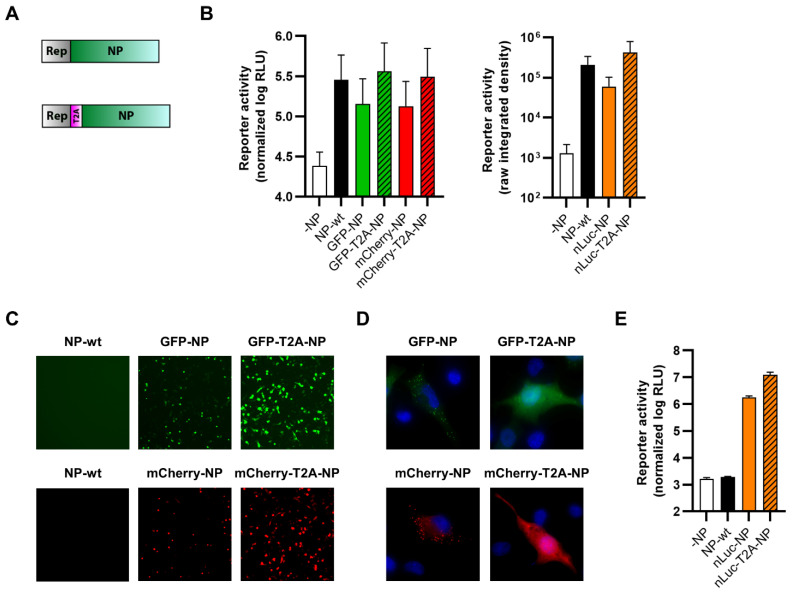
N-terminal fusion of TCRV NP to reporter proteins. (**A**) Schematic. Reporter proteins were N-terminally fused directly to TCRV NP (top) or separated by a T2A site (bottom). (**B**) Minigenome activity. BSR-T7/5 cells were transfected with plasmid components for either a nLuc-based minigenome assay (left) or a GFP-based minigenome assay (right). For nLuc-based minigenome assays (left), cells were lysed after 48 h and both nLuc and FLuc activities were measured. Results are shown as the mean ± SD of the log of nLuc activity normalized to FLuc activity from three independent experiments. For GFP-based minigenome assays (right), pictures of each well were taken and used to measure the raw integrated density in the GFP channel. Results are shown as the mean ± SD of four independent experiments. (**C**) Reporter activity (GFP/mCherry). Pictures of cells transfected as in (**B**) were taken 48 h post-transfection at 10× magnification. Results are shown as representative images from one experiment out of two independent experiments. (**D**) Reporter localization. Pictures of cells transfected as in (**B**) were taken 24 h post-transfection at 63× magnification after fixation and DAPI staining. Results are shown as representative images from one experiment out of two independent experiments. (**E**) Reporter activity (nLuc). Cells transfected as in (**B**) were lysed 48 h post-transfection, and the nLuc activity in lysates was measured. Results are shown as mean ± SD of four independent experiments.

Next, we sought to generate viruses where the reporter proteins were fused either directly or separated by a T2A self-cleavage site to the different viral proteins. Such 2A sequences cause ribosomal skipping during translation, which is responsible for the apparent cleavage between the reporter and viral proteins [57]. We first fused our different reporter proteins N-terminally to TCRV NP (Figure 2A) in the context of expression plasmids in order to test their functionality, as measured based on their ability to support viral RNA synthesis in a minigenome assay. Specifically, the functionality of GFP and mCherry constructs was assessed using a nLuc-based minigenome (Figure 2B, left panel), while the functionality of nLuc constructs was assessed using a GFP-based minigenome (Figure 2B, right panel). These data showed that, while T2A-fused constructs showed levels of activity similar to unmodified wild-type TCRV NP (NP-wt), the directly-fused constructs were notably impaired (Figure 2B). This was despite the fact that all the tested constructs displayed robust reporter activity following transfection (Figure 2C–E). Interestingly, while the expression of the directly N-terminally fused NP reporter constructs appears to be somewhat lower when observed under low magnification (Figure 2C), this may be due to differences in the localization of the directly and T2A-fused reporter proteins. Specifically, we saw that T2A-fused fluorescent reporters had a cytoplasmic distribution, consistent with their cleavage from NP and resulting in a typical diffuse localization of these fluorescent proteins (Figure 2D, right panels). In contrast, directly-fused NP constructs displayed a punctate pattern, characteristic of inclusion bodies, which are known to be formed by TCRV NP [58] (Figure 2D, left panels).

**Figure 3:**
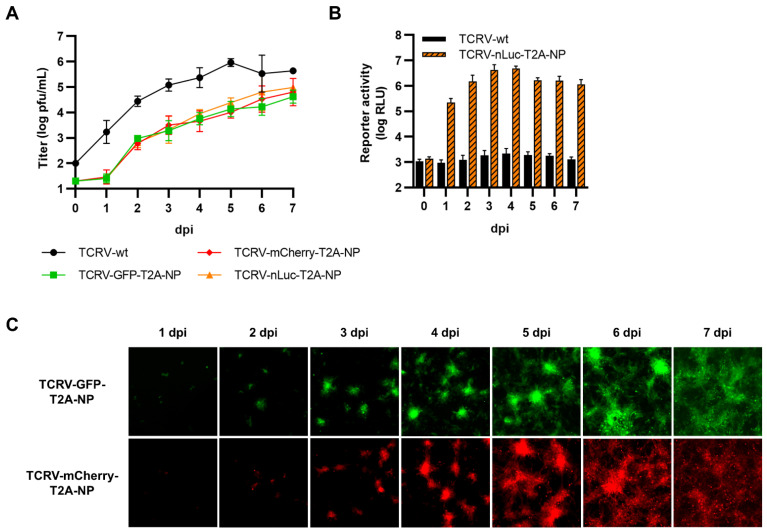
Recombinant viruses encoding N-terminal fusions of TCRV NP to reporter proteins. (**A**) Growth kinetics. Vero76 cells were infected at MOI = 0.005 with the indicated viruses. Supernatants were harvested on the indicated days and titrated by plaque assay in Vero76 cells. Results are shown as mean ± SD of three independent experiments. (**B**) Reporter activity (nLuc). Cells infected as in (**A**) were lysed on the indicated days, and nLuc activity in lysates was measured. Results are shown as mean ± SD of three independent experiments. (**C**) Reporter activity (GFP/mCherry). Pictures of cells infected as in (**A**) were taken on the indicated days at 10× magnification. Results are shown as representative images from one experiment out of three independent experiments.

While we were able to rescue viruses expressing each of the three reporters fused via T2A to the N-terminus of NP, the same was not possible for directly fused constructs, consistent with their impaired minigenome activity. Evaluation of the growth kinetics of these viruses in Vero76 cells showed that they were all markedly attenuated compared to TCRV-wt, and that the degree of attenuation observed was the same regardless of the reporter protein used (Figure 3A). Robust reporter expression was also observed from our N-terminally T2A-fused NP viruses regardless of which reporter was used (Figure 3B,C). Specifically, and similar to the data with the trisegmented TCRV approach, we saw clear expression of the nLuc reporter starting already from 1 dpi (Figure 3B), while for the fluorescent reporters, this was the case only from 2 dpi (Figure 3C). Overall, while direct fusion of reporters at the N-terminus of TCRV NP was unsuccessful, an approach that incorporates a T2A self-cleavage site was both viable and resulted in strong reporter activity that appears to be relatively independent of the reporter used.

**Figure 4:**
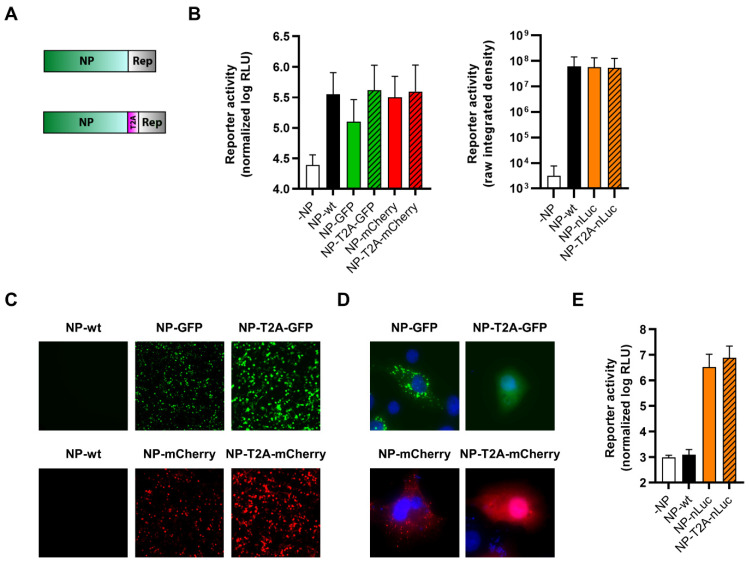
C-terminal fusion of TCRV NP to reporter proteins. (**A**) Schematic. Reporter proteins were C-terminally fused directly to TCRV NP (top) or separated by a T2A site (bottom). (**B**) Minigenome activity. BSR-T7/5 cells were transfected with plasmid components for either a nLuc-based minigenome assay (left), or a GFP-based minigenome assay (right). For nLuc-based minigenome assays (left), cells were lysed after 48 h and both nLuc and FLuc activities were measured. Results are shown as the mean ± SD of the log of nLuc activity normalized to FLuc activity from five independent experiments. For GFP-based minigenome assays (right), pictures of each well were taken and used to measure the raw integrated density in the GFP channel. Results are shown as the mean ± SD of two independent experiments. (**C**) Reporter activity (GFP/mCherry). Pictures of cells transfected as in (**B**) were taken 48 h post-transfection at 10× magnification. Results are shown as representative images from one experiment out of two independent experiments. (**D**) Reporter localization. Pictures of cells transfected as in (**B**) were taken 24 h post-transfection at 63× magnification after fixation and DAPI staining. Results are shown as representative images from one experiment out of two independent experiments. (**E**) Reporter activity (nLuc). Cells transfected as in (**B**) were lysed 48 h post-transfection, and nLuc activity in lysates was measured. Results are shown as mean ± SD of three independent experiments.

We also used the same approach to generate plasmids expressing TCRV NP C-terminally fused to each of the reporter proteins, with or without a T2A self-cleavage site (Figure 4A). When these constructs were tested for their functionality in a minigenome assay, all of them were able to support minigenome activity to a similar level than NP-wt, with the exception of NP directly fused to GFP, which was markedly impaired in its ability to support viral RNA synthesis (Figure 4B). Consistent with this, all C-terminally fused NP constructs displayed significant reporter activity following transfection (Figure 4C–E), and when either GFP or mCherry were directly fused to NP, they could be observed to be localized in punctate formations reminiscent of inclusion bodies, as opposed to the cleavable reporters, which were found diffusely distributed throughout the cytoplasm (Figure 4D). However, despite their high degree of functionality in the MG assay (in most cases) and apparently authentic intracellular distribution of the reporter-fused NP proteins, we were unable to rescue any of the corresponding viruses.

### 3.3. Reporter Fusions with TCRV GPC

**Figure 5:**
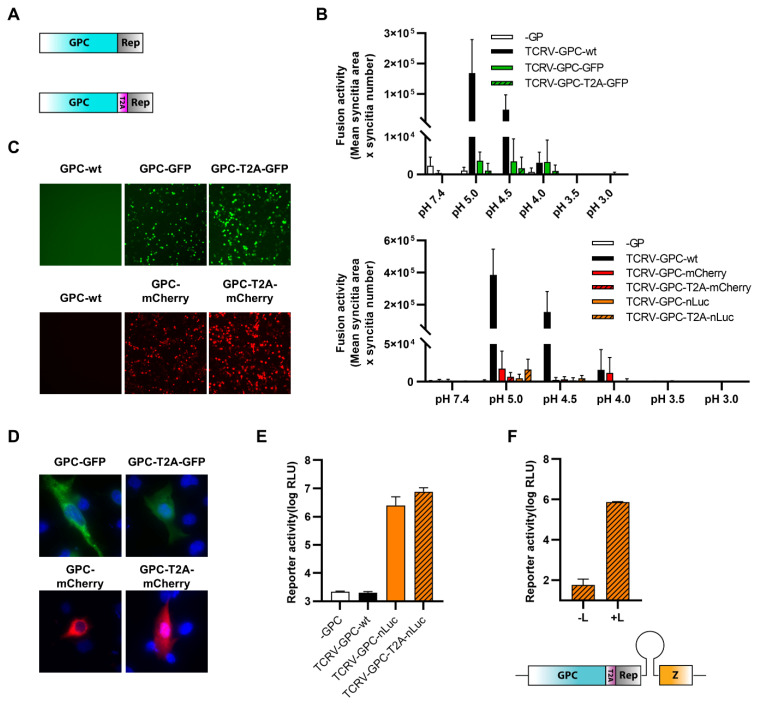
C-terminal fusion of TCRV GPC to reporter proteins. (**A**) Schematic. Reporter proteins were C-terminally fused directly to TCRV GPC (top) or separated by a T2A site (bottom). (**B**) Fusion activity. BSR-T7/5 cells were transfected with plasmids encoding mCherry, and GPC constructs fused to GFP (top) or constructs encoding GFP, and GPC constructs fused to either mCherry or nLuc (bottom). After 24 h, cells were treated at the indicated pH for 5 min at 37 °C and rinsed. Cells were fixed 24 h later and syncytia formation was measured. Results are shown as the mean ± SD of the total syncytia area from ten random fields of view from three independent experiments. (**C**) Reporter activity (GFP/mCherry). Pictures of cells transfected as in (**B**) were taken 48 h post-transfection at 10× magnification. Results are shown as representative images from one experiment out of two independent experiments. (**D**) Reporter localization. Pictures of cells transfected as in (**B**) were taken 24 h post-transfection at 63× magnification after fixation and DAPI staining. Results are shown as representative images from one experiment out of two independent experiments. (**E**) Reporter activity (nLuc). Cells transfected as in (**B**) were lysed 48 h post-transfection, and nLuc activity in lysates was measured. Results are shown as mean ± SD of three independent experiments. (**F**) trVLP entry. BSR-T7/5 cells were transfected with plasmids encoding the T7 polymerase, TCRV L, TCRV NP, and FLuc (as a transfection control), in addition to a plasmid encoding for the bicistronic minigenome. trVLPs were harvested 72 h post-transfection and were used to infect Huh7 cells that were transfected with plasmids encoding for TCRV L and TCRV NP 24 h before infection. Cells were lysed 72 h postinfection, and nLuc activity was measured. Results are shown as the mean ± SEM of the log of nLuc activity from two independent experiments (top). A schematic of the bicistronic S-segment-based minigenome, in which the NP ORF was replaced by GPC fused at the C-terminus to a T2A cleavable nLuc and the GPC ORF was replaced by TCRV Z, is shown (bottom).

Due to the presence of the SSP at the N-terminus of GPC, which needs to be correctly translocated and post-translationally processed, we could only fuse reporter proteins to GPC C-terminally (Figure 5A). To assess the functionality of these reporter-fused GPCs, we first performed cell–cell fusion assays to measure the ability of mature TCRV GP complexes to trigger fusion of plasma membranes between adjacent cells. For this, cells were transfected both with expression plasmids for the respective reporter-fused GPC constructs as well as a fluorescent reporter protein (i.e., GFP or mCherry depending on the GPC construct being analyzed) for better visualization of the syncytia. Fusion was then triggered by low-pH treatment, and syncytia formation was monitored by fluorescence microscopy. While GP-wt displayed significant fusion activity at pH 5, the reporter-fused constructs were strongly impaired (Figure 5B), despite displaying reporter activity following transfection (Figure 5C–E). At higher magnification, we could also observe that, for the directly fused constructs, most of the expressed GP appeared to be retained intracellularly in compartments that could correspond to the ER/Golgi network (Figure 5D). Surprisingly, however, we found that results differed when we examined the activity of GPC with a cleavable C-terminal fusion to nLuc using a trVLP assay. This assay was based on a bicistronic S-segment-based minigenome, in which the NP ORF was replaced by GPC fused at the C-terminus to a T2A cleavable nLuc and the GPC ORF was replaced by TCRV Z (Figure 5F, bottom). Using this approach, we showed that the GPC-T2A-nLuc construct was still capable of supporting robust production of trVLPs that were able to infect fresh target cells (Figure 5F, top), suggesting that a biologically meaningful level of activity may still be retained by these constructs during cell entry. Nonetheless, we were unable to rescue any of these viruses, correlating with their reduced capacity for fusion, as seen in the cell–cell fusion assay.

### 3.4. Reporter Fusions with TCRV Z

**Figure 6:**
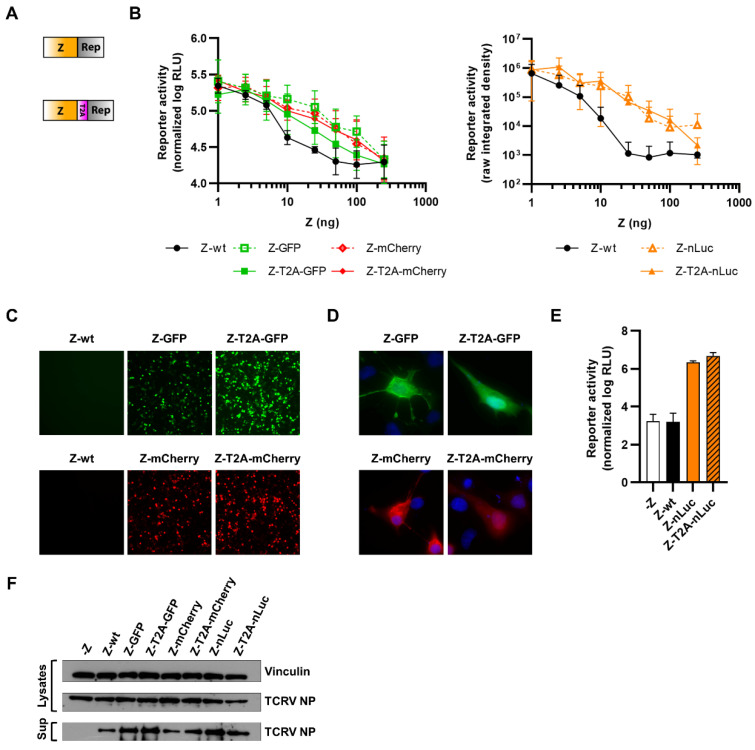
C-terminal fusion of TCRV Z to reporter proteins. (**A**) Schematic. Reporter proteins were C-terminally fused directly to TCRV Z (top) or separated by a T2A site (bottom). (**B**) Minigenome activity. BSR-T7/5 cells were transfected with plasmid components for either a nLuc-based minigenome assay (left), or a GFP-based minigenome assay (right), alongside increasing amounts of TCRV Z constructs fused to GFP/mCherry or nLuc, respectively. For nLuc-based minigenome assays (left), cells were lysed after 48 h and both nLuc and FLuc activities were measured. Results are shown as the mean ± SD of the log of nLuc activity normalized to FLuc activity from three independent experiments. For GFP-based minigenome assays (right), pictures of each well were taken and used to measure the raw integrated density in the GFP channel. Results are shown as the mean ± SD of two independent experiments. (**C**) Reporter activity (GFP/mCherry). Pictures of cells transfected as in (**B**) and with 250 μg of the indicated constructs expressing TCRV Z were taken 48 h post-transfection at 10× magnification. Results are shown as representative images from one experiment out of two independent experiments. (**D**) Reporter localization. Pictures of cells transfected as in (**C**) were taken 24 h post-transfection at 63× magnification after fixation and DAPI staining. Results are shown as representative images from one experiment out of two independent experiments. (**E**) Reporter activity (nLuc). Cells transfected as in (**C**) were lysed 48 h post-transfection, and nLuc activity in lysates was measured. Results are shown as mean ± SD of three independent experiments. (**F**) VLP budding mediated by TCRV Z reporter proteins. HEK293T cells were transfected with pCAGGS expressing TCRV NP-Flag and TCRV GPC-Flag with or without TCRV Z, or the indicated TCRV Z reporter constructs. After 4 days, VLP-containing supernatants (sup) were harvested and purified over a 20% sucrose cushion while the cells were lysed in 1% Triton X-100. The resulting samples were analyzed by Western blot for TCRV NP release and expression, respectively. Vinculin expression in the lysates was used as a loading control.

Due to the presence of a myristoylation site at position G2 that is critical for Z budding activity [59], we were also only able to generate Z with C-terminally fused reporters (Figure 6A). Since Z has been shown to inhibit the activity of the viral polymerase L, in order to regulate the switch from replication to budding [6], we tested the function of our constructs by assessing their ability to inhibit minigenome activity. For this, we transfected cells with the standard minigenome components, alongside increasing amounts of the various Z constructs. We found that all of the constructs were able to strongly inhibit minigenome activity, albeit to a slightly lower extent than Z-wt (Figure 6B). Further, all constructs displayed reporter activity (Figure 6C–E) and, importantly, the constructs where Z was directly fused to GFP or mCherry were found to be mainly localized at the plasma membrane, consistent with the expected distribution of the Z protein (Figure 6D). 

Importantly, however, arenavirus Z is a highly multifunctional protein, and one of its other major roles is to drive particle budding. Therefore, in order to also evaluate the budding efficiency of our TCRV Z reporter constructs, we performed a budding assay. For this, cells were transfected with TCRV Z, NP-Flag, and GPC-Flag in order to produce virus-like particles (VLPs), which were then purified over a sucrose cushion and analyzed by Western blot for TCRV NP. All TCRV Z constructs were able to mediate the production of VLPs incorporating NP at least as efficiently as Z-wt, while GFP and nLuc constructs appeared to be able to drive VLP budding even more efficiently than Z-wt (Figure 6F).

**Figure 7:**
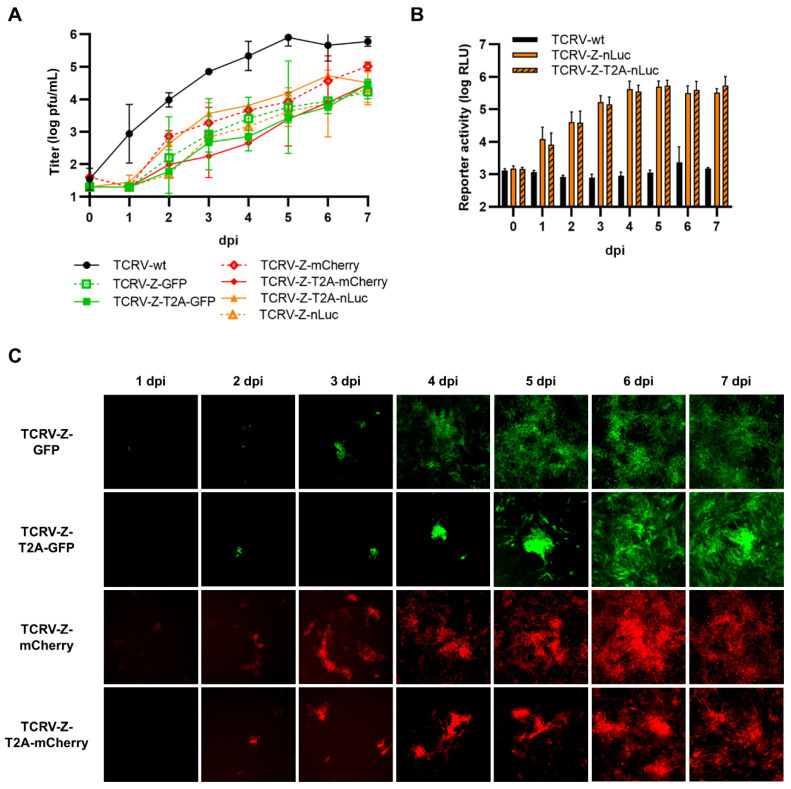
Recombinant viruses encoding C-terminal fusions of TCRV Z to reporter proteins. (**A**) Growth kinetics. Vero76 cells were infected at MOI = 0.005 with the indicated viruses. Supernatants were harvested on the indicated days and titrated by plaque assays in Vero76 cells. Results are shown as mean ± SD of two independent experiments. (**B**) Reporter activity (nLuc). Cells infected as in (**A**) were lysed on the indicated days, and nLuc activity in lysates was measured. Results are shown as mean ± SD of three independent experiments. (**C**) Reporter activity (GFP/mCherry). Pictures of cells infected as in (**A**) were taken on the indicated days at 10× magnification. Results are shown as representative images from one experiment out of three independent experiments.

Recombinant viruses corresponding to each of the C-terminal Z reporter fusions, both direct and T2A cleavable, could be rescued and grew efficiently, although they were still attenuated (Figure 7A). Surprisingly, viruses encoding Z directly fused to GFP or mCherry were less attenuated that their T2A counterparts, while we observed the opposite for nLuc-expressing viruses (Figure 7A). All TCRV Z-reporter viruses displayed reporter activity (Figure 7B,C); however, for fluorescent viruses, the reporter activity was comparatively weak (i.e., in comparison to what was observed for viruses encoding NP fused to the same reporters) and could only be readily observed starting at 3–4 dpi (Figure 7C). In contrast, clear accumulation of nLuc reporter activity could still be seen with these viruses starting as early as 1 dpi (Figure 7B).

### 3.5. Reporter Fusions with TCRV L

**Figure 8:**
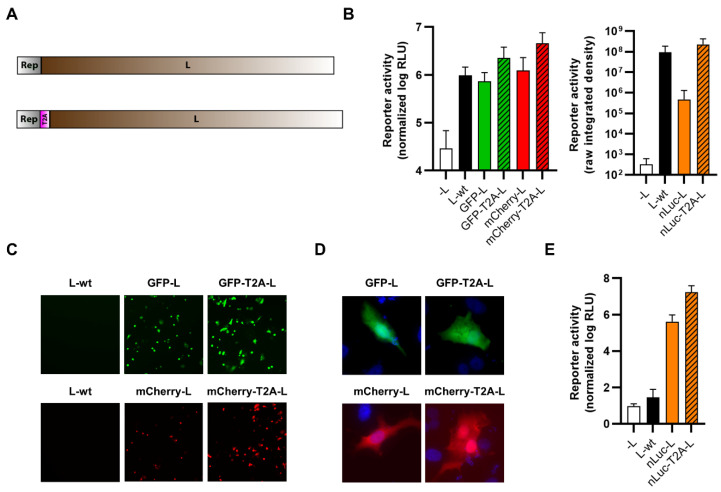
N-terminal fusion of TCRV L to reporter proteins. (**A**) Schematic. Reporter proteins were N-terminally fused directly to TCRV L (top) or separated by a T2A site (bottom). (**B**) Minigenome activity. BSR-T7/5 cells were transfected with plasmid components for either a nLuc-based minigenome assay (left), or a GFP-based minigenome assay (right). For nLuc-based minigenome assays (left), cells were lysed after 48 h and both nLuc and FLuc activities were measured. Results are shown as the mean ± SD of the log of nLuc activity normalized to FLuc activity from three independent experiments. For GFP-based minigenome assays (right), pictures of each well were taken and used to measure the raw integrated density in the GFP channel. Results are shown as the mean ± SD of three independent experiments. (**C**) Reporter activity (GFP/mCherry). Pictures of cells transfected as in (**B**) were taken 48 h post-transfection at 10× magnification. Results are shown as representative images from one experiment out of two independent experiments. (**D**) Reporter localization. Pictures of cells transfected as in (**B**) were taken 24 h post-transfection at 63× magnification after fixation and DAPI staining. Results are shown as representative images from one experiment out of two independent experiments. (**E**) Reporter activity (nLuc). Cells transfected as in (**B**) were lysed 48 h post-transfection, and nLuc activity in lysates was measured. Results are shown as mean ± SD of three independent experiments.

Lastly, we generated reporter fusions with TCRV L using various approaches. First, we generated expression plasmids with the reporters, with or without a T2A self-cleavage site, N-terminally fused to L (Figure 8A). All of the resulting constructs were able to support robust minigenome activity, although the directly fused constructs appeared to be slightly impaired compared with wild-type (Figure 8B). We also observed reporter activity for all constructs (Figure 8C–E). Similar to the constructs with a T2A self-cleavage site, the constructs with GFP or mCherry directly fused to L did not show any specific localization, but rather, the reporter was diffusely localized throughout the cytoplasm (Figure 8D). Despite their functionality in a minigenome system, we were unable to rescue any of the corresponding viruses encoding N-terminal reporter fusions to L, regardless of whether a T2A self-cleavage site was included or not.

**Figure 9:**
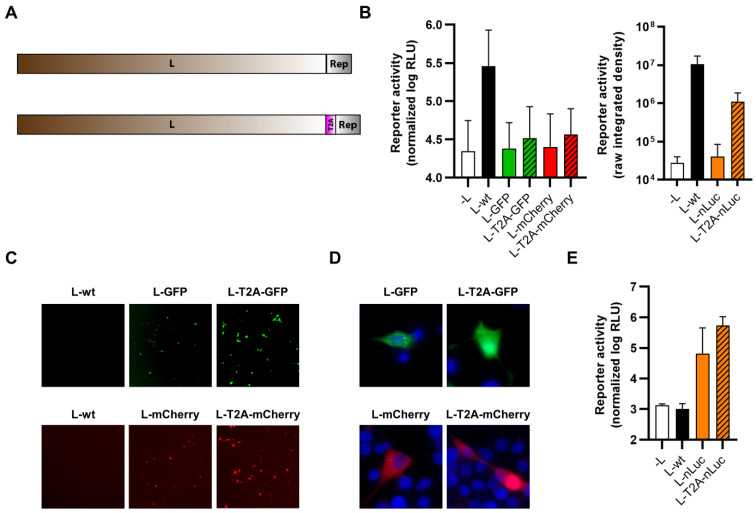
C-terminal fusion of TCRV L to reporter proteins. (**A**) Schematic. Reporter proteins were C-terminally fused directly to TCRV L (top) or separated by a T2A site (bottom). (**B**) Minigenome activity. BSR-T7/5 cells were transfected with plasmid components for either a nLuc-based minigenome assay (left), or a GFP-based minigenome assay (right). For nLuc-based minigenome assays (left), cells were lysed after 48 h and both nLuc and FLuc activities were measured. Results are shown as the mean ± SD of the log of nLuc activity normalized to FLuc activity from two independent experiments. For GFP-based minigenome assays (right), pictures of each well were taken and used to measure the raw integrated density in the GFP channel. Results are shown as the mean ± SD of two independent experiments. (**C**) Reporter activity (GFP/mCherry). Pictures of cells transfected as in (**B**) were taken 48 h post-transfection at 10× magnification. Results are shown as representative images from one experiment out of two independent experiments. (**D**) Reporter localization. Pictures of cells transfected as in (**B**) were taken 24 h post-transfection at 63× magnification after fixation and DAPI staining. Results are shown as representative images from one experiment out of two independent experiments. (**E**) Reporter activity (nLuc). Cells transfected as in (**B**) were lysed 48 h post-transfection, and nLuc activity in lysates was measured. Results are shown as mean ± SD of two independent experiments.

Next, we used the same approach to generate TCRV L constructs with C-terminally fused reporters, again with or without a T2A self-cleavage site (Figure 9A). However, we observed that all expression constructs had strongly impaired minigenome activity similar to background levels without L (Figure 9B). This was despite both types of constructs showing reporter activity (Figure 9C–E). Similar to the N-terminal fusions with L, reporter proteins were found to be diffusely localized throughout the cytoplasm regardless of whether a T2A self-cleavage site was included or not. Due to the total lack of activity in the minigenome system, we did not attempt to rescue the corresponding viruses.

**Figure 10:**
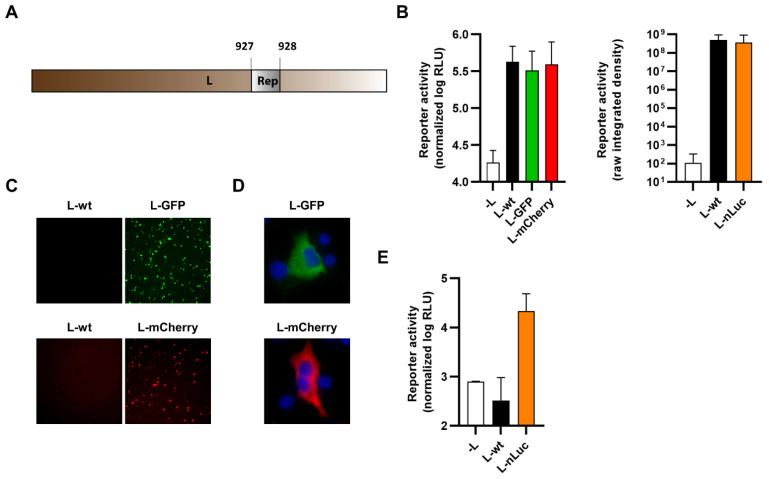
Internal fusion of TCRV L to reporter proteins. (**A**) Schematic. Reporter proteins were inserted within the TCRV L ORF between amino acids 927 and 928. (**B**) Minigenome activity. BSR-T7/5 cells were transfected with plasmid components for either a nLuc-based minigenome assay (left) or a GFP-based minigenome assay (right). For nLuc-based minigenome assays (left), cells were lysed after 48 h and both nLuc and FLuc activities were measured. Results are shown as the mean ± SD of the log of nLuc activity normalized to FLuc activity from four independent experiments. For GFP-based minigenome assays (right), pictures of each well were taken and used to measure the raw integrated density in the GFP channel. Results are shown as the mean ± SD of four independent experiments. (**C**) Reporter activity (GFP/mCherry). Pictures of cells transfected as in (**B**) were taken 48 h post-transfection at 10× magnification. Results are shown as representative images from one experiment out of two independent experiments. (**D**) Reporter localization. Pictures of cells transfected as in (**B**) were taken 24 h post-transfection at 63× magnification after fixation and DAPI staining. Results are shown as representative images from one experiment out of two independent experiments. (**E**) Reporter activity (nLuc). Cells transfected as in (**B**) were lysed 48 h post-transfection, and nLuc activity in lysates was measured. Results are shown as mean ± SD of four independent experiments.

Finally, we identified a region of low conservation within arenavirus polymerase sequences (Supplementary Figure S1), which presumably corresponds to a flexible hinge region such as has been described for the polymerases of other NSVs. This region was then exploited to internally fuse reporter proteins within TCRV L at a position between amino acids 927–928 (Figure 10A). Expression constructs were tested for their functionality in a minigenome assay with all constructs supporting minigenome activity at a level similar to wild-type L (Figure 10B). The constructs also all displayed reporter activity (Figure 10C–E) although, as observed for terminal fusions with the L protein, no specific localization of the fluorescent reporters could be observed (Figure 10D). 

**Figure 11:**
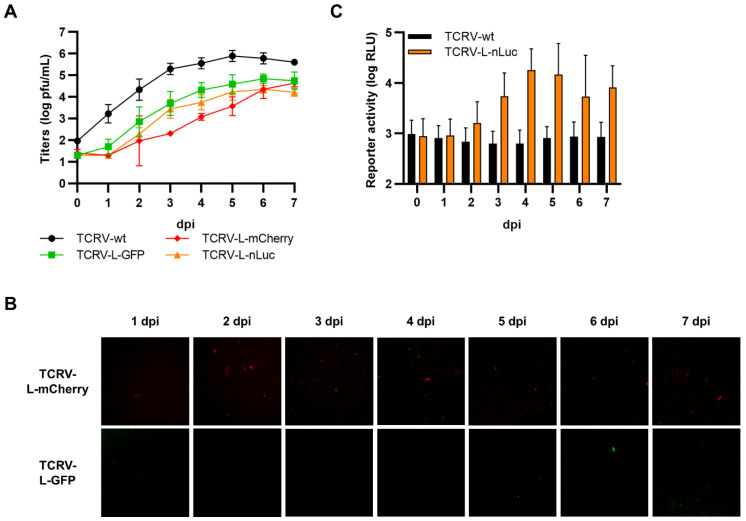
Recombinant viruses encoding internal fusions of TCRV L to reporter proteins. (**A**) Growth kinetics. Vero76 cells were infected at MOI = 0.005 with the indicated viruses. Supernatants were harvested on the indicated days and titrated by plaque assays in Vero76 cells. Results are shown as mean ± SD of three independent experiments. (**B**) Reporter activity (GFP/mCherry). Pictures of cells infected as in (**A**) were taken on the indicated days at 10× magnification. Results are shown as representative images from one experiment out of two independent experiments. (**C**) Reporter activity (nLuc). Cells infected as in (**A**) were lysed on the indicated days, and nLuc activity in lysates was measured. Results are shown as mean ± SD of four independent experiments.

Viruses encoding internal L fusions with each of the three reporters (i.e., GFP, mCherry, and nLuc) were successfully rescued and could efficiently replicate, although they were modestly attenuated compared with TCRV-wt (Figure 11A). However, we were unable to detect fluorescence activity for either the GFP or the mCherry virus (Figure 11B), despite confirming the integrity of the reporter fusion in the rescued viruses. In contrast, the nLuc-expressing virus displayed increasing reporter activity over time, starting at 2–3 dpi, but the values remained low in comparison to those obtained using other reporter fusion strategies (Figure 11C).

## 4. Discussion

The availability of reporter-expressing viruses greatly facilitates several areas of virus research, including diagnostics, drug testing, and host factor identification, where they are particularly beneficial in high-throughput screening-based approaches. They are also indispensable tools for studies focused on virus imaging both in vitro and in vivo. However, the incorporation of foreign genes, such as reporters, is well-known to influence virus growth to a greater or lesser extent depending on the incorporation strategy and the reporter used [60,61,62,63]. At the same time, the method by which the reporter is inserted can also affect the expression level of the reporter, and thus, the sensitivity with which virus infection can be detected [38,39,40,63,64,65]. Further, the decision whether to link the reporter directly to a viral protein, express it as a cleavable fusion, or even as a completely separate gene, impacts the localization of the reporter within the cell. To what extent these considerations are a concern is highly application-dependent. For instance, for in vivo applications, virus attenuation is a key consideration, while for in vitro screening approaches where an effect (e.g., inhibition or knock-down) has to be maintained, reporter brightness may play a more significant role because of its impact on assay time. In contrast, for some imaging-based studies, an authentic localization of the reporter, consistent with the normal distribution and function of the viral protein it is linked to, is the primary consideration. In contrast, for diagnostic and screening applications, reporter distribution is likely to be irrelevant in most cases, as the goal here is often simply to obtain an easier read-out. Ultimately, the choice of reporter, and particularly whether it is fluorescent or luminescent, also greatly influences which applications a given reporter virus will be suitable for, and is a particularly important consideration where highly specialized equipment is required, e.g., for high-content imaging. Given all these different needs, and especially in light of the challenges that tend to be encountered in developing a “perfect” reporter virus that is not attenuated and yet delivers robust reporter expression, it is often necessary to have a variety of tools available based on different approaches. Unfortunately, until now, only very few strategies for creating reporter-expressing arenaviruses have been considered. As such, this study took a systematic approach to the generation of reporter-expressing TCRVs, and in doing so, identified several additional strategies for the generation of fluorescent and luminescent fusions with arenavirus proteins, some of which were also viable as recombinant viruses (summarized in Table 1).

Given their unusual ambisense genome arrangement, the introduction of reporter genes into the arenavirus genome is challenging. For instance, unlike for mononegaviruses, reporter proteins cannot simply be introduced as additional transcriptional units at various positions within the genome [61,62,63,65,66]. As such, the first approaches to successfully generate a reporter-expressing arenavirus focused on creating trisegmented viruses in which there are 2 copies of the S-segment—one in which the NP gene is deleted and one in which the GP gene is deleted—thus creating additional coding capacity for either one or two genes of interest [38]. This approach relies on the fact that, unlike influenza viruses, for which packaging of the individual segments is tightly orchestrated by noncoding regions and parts of the terminal coding regions [67], arenavirus segment packaging appears to be less restrictive and allows for packaging of multiple copies of the same segment [38,68]. This approach has since been used to successfully rescue several arenaviruses expressing various genes of interest [38,39,41,69], including most recently a combined GFP and Gaussia luciferase-expressing TCRV [41]. It is, therefore, unsurprising that we could also rescue trisegmented TCRV viruses expressing various fluorescent or luminescent reporter proteins from a duplicated S-segment as a part of this study. Consistent with previous studies, these trisegmented TCRV viruses were all modestly attenuated [41]. Further, our data using different insertion positions and reporters also suggest that both the size and location of the reporter genes have an impact on virus growth. Specifically, we found that virus growth was less impaired in trisegmented TCRVs where the reporter gene was inserted in the GPC ORF, compared with those where it was inserted in the NP ORF. Further, we found that using a shorter reporter gene (e.g., nLuc) appears to have a stronger negative impact on virus growth. Interestingly, this is in contrast with the results of a previous study using LCMV, which found that virus growth was more profoundly impaired when longer reporter genes (i.e., FLuc) were used [38], suggesting that other aspects of the sequence or genome context may also play a role. However, consistent with a previous study using trisegmented GFP- and CAT-expressing LCMV, we also observed differences in reporter expression from the different ORFs, with stronger reporter activity seen when the reporter genes were inserted in the NP ORF compared with the GPC ORF [38]. This could be at least partly due to the delayed transcription, and thus, protein expression, of the GP ORF as a result of the ambisense coding strategy used by arenaviruses.

The only other approach that had been previously reported focused on the possibility of fusing a reporter protein to the N-terminus of NP. To avoid potential negative effects of a direct fusion, these approaches used a P2A (porcine teschovirus-1) self-cleavage site to separate the viral and reporter proteins [35,36,37,41,44]. This approach has been shown to be successful for LASV [37], LCMV [35,44], Lujo virus [36], and TCRV [41], but has the notable disadvantage that, while it allows infection of cells to be visualized, it does not provide localization information about the viral protein to which it was fused. Consistent with these studies, we were also able to rescue TCRV with reporters N-terminally fused to NP via a T2A self-cleavage site. Interestingly, however, these T2A fused viruses showed reduced growth compared with wild-type, which is in contrast with the previously published work using LASV [37], Lujo virus [36], and TCRV [41] where no such attenuation was observed. For LCMV, only a slight attenuation was observed [35,44]. Since all of these previous studies used a P2A sequence (rather than a T2A sequence), this suggests that this may be a key factor. In our study, T2A was selected based on a study of the cleavage efficiency of 2A sequences that suggested that T2A sequences are the most efficiently processed following in vitro translation [70]. However, the results using these different sequences in the context of reporter-expressing arenaviruses appear to rather support another study that suggested that the P2A site might be more efficient at mediating cleavage in some cell culture contexts [71]. As such, the use of P2A sequences may indeed be preferred for reporter virus construction, and may also be an approach to further improving this and other strategies we have developed as part of this study. Nonetheless, both the fluorescent and luminescent versions of this construct show strong reporter expression that accumulates rapidly postinfection and, thus, are suitable for a wide range of applications where modestly decreased growth is not a critical concern.

Building on these previous approaches, the major goal of this work was to expand the range of strategies available for generating reporter-expressing arenaviruses in order to develop resources that might be suitable for additional kinds of studies (e.g., those where imaging of protein localization is important) that are not currently possible using the existing strategies. Consequently, we were interested in exploring both the possibility of creating fusions with other viral proteins, as well as generating noncleavably fused viral proteins that might be suitable for imaging applications, including live cell imaging. Unfortunately, unlike the T2A-linked versions, we could not rescue TCRV containing NP directly fused to reporters at the N-terminus. Since these reporter-fused proteins also showed decreased functionality in the minigenome system, we suggest that this might be due to impairment of NP–NP interactions, which are mediated by the N-terminal domain of the protein, and are known to be important for RNA binding and, subsequently, replication and transcription [72]. In contrast, C-terminal fusion of reporters to NP had little impact on minigenome activity, with the exception of direct fusions to GFP. Since similar issues were not observed using a direct mCherry fusion, we suggest that this problem may be due to the tendency of GFP to dimerize (whereas mCherry is monomeric), which may then also impair the normal oligomerization of NP. However, despite their functionality in viral RNA synthesis, we still could not rescue any of the viruses with C-terminal NP fusions, regardless of whether they were directly or cleavably fused. This suggests that the presence of a reporter protein (or even the residual short amino acid sequence left following T2A cleavage) at this location might impair the ability of NP to undergo other protein–protein interactions. In particular, the C-terminal domain of NP is responsible for interaction with Z, which impacts both budding efficiency and recruitment of RNPs to the budding particles [45,73,74], and we cannot currently exclude perturbations in this interaction. Nonetheless, despite not being viable in a virus context, these directly C-terminally reporter fused NPs are well-expressed and easily detectable, and thus, still have significant potential value, for instance in imaging studies examining inclusion body dynamics and/or protein trafficking. In these cases, the fluorescent versions of NP could be transfected either alone, together with other components of the ribonucleoprotein complex, or even together with a TCRV-wt infection, such as has been previously done for studies of LCMV Z trafficking [75].

When we examined the possibility of generating functional reporter fusions to the C-terminus of GPC, cell–cell fusion assays clearly showed that both cleavable and direct GPC fusions strongly impaired fusion activity, independent of the reporter used. Interestingly, however, we were still able to produce trVLPs that efficiently entered target cells when using a C-terminally fused GPC-T2A-nLuc. One possible explanation for this difference is that the presence of additional sequences at the C-terminus of the GP2 fusion-mediating subunit might stabilize the prefusion conformation of the glycoprotein, such that it requires an authentic late endosome membrane lipid and/or protein composition to fuse efficiently. Indeed, specific lipids have been shown to facilitate the fusion of arenaviruses [76], while intracellular receptors such as Lamp1 and CD63 have been shown to lower the pH requirement for viral fusion of LASV and Lujo virus, respectively [77,78]. However, regardless of the mechanistic basis, this ability to create even cleavable fusions between luciferase and GPC opened up the possibility to make improvements to the trVLP approach based on the construction of multicistronic minigenomes (i.e., those where the viral proteins associated with morphogenesis/budding are expressed from the minigenome itself). Such an approach reduces assay complexity (and thereby potentially increases assay robustness) by eliminating the need for multiple transfections, while at the same time allowing the possibility to regulate the expression of these genes, which are often inhibitory for viral RNA synthesis, as is the case with the arenavirus Z [6]. Similar multicistronic trVLP systems for filoviruses that have been developed in recent years have shown that these systems are dramatically more efficient than monocistronic minigenome-based systems [79], and our data indicate that this is also the case for arenaviruses. Indeed, while we were previously able to develop a classical monocistronic minigenome-based trVLP system for JUNV [16], a similar approach for TCRV was unsuccessful (data not shown), while the corresponding multicistronic approach used in this study proved very efficient. The availability of this additional reverse genetics-based tool for TCRV has clear potential applications for mechanistic studies, since such trVLPs are based exclusively on arenavirus components and, thus, can be expected to mediate entry and fusion in a mechanistically authentic way. Nonetheless, despite the ability of at least cleavable fusion-based approaches to mediate entry in trVLP-based approaches, we were unable to rescue any recombinant viruses expressing reporters fused to GPC, suggesting that there is indeed some degree of functional impairment compared with wild-type even during entry in an authentic cellular context. Therefore, rather than focusing on C-terminal fusions, future strategies might rather consider adding the reporter at the cleavage site between GP1 and GP2.

Despite challenges in generating fusions, and particularly direct fusions, to either NP or GP we were, somewhat surprisingly, able to rescue recombinant TCRVs encoding both direct and cleavable C-terminal reporter fusions to Z. Further, while intuitively, one might expect shorter sequences to be less disruptive, we observed that direct fusions of fluorescent proteins to Z resulted in viruses that replicated slightly more efficiently than their T2A-site-containing counterparts (where only a short peptide sequence remains following reporter cleavage). This is despite the fact that the reporters used are relatively long (171–239 amino acids) in comparison to the protein itself (95 amino acids) and that Z needs to undergo not only extensive homo-oligomerization, but also protein–protein interactions with NP, GP, and L to fulfill its diverse functions in the virus lifecycle. Interestingly, these differences in viral growth between the directly fused constructs and the corresponding constructs containing a T2A site do not seem to be explained by differences in budding efficiency or the interactions of Z with either NP or L, as seen from the VLP and minigenome data. While plasmid-based expression of LCMV Z directly fused to RFP at its C-terminus has been previously used in microscopy studies exploring the trafficking of this protein [75], this is the first indication that such fusions are also viable in a viral context. It is, however, worth noting that budding of TCRV Z is somewhat unusual among arenaviruses, in that it occurs without canonical proline-rich late domains that normally play a major role in the recruitment of ESCRT proteins [80]. Further, it requires support from NP for efficient budding [45], suggesting that TCRV NP might also be playing a role in the recruitment of the ESCRT machinery [7]. As such, it will be interesting to determine whether these specific features of TCRV budding confer increased tolerance of TCRV Z to C-terminal reporter fusions, or whether this strategy is viable for other arenaviruses as well.

In contrast to our relative success creating fusions to Z, for TCRV L this proved to be more challenging. In general, neither direct nor T2A cleavable N-terminal fusions interfered with the ability of L to support minigenome activity (with the exception of the direct fusion with nLuc), and indeed, cleavable fusions even seem to slightly enhance minigenome activity. Nonetheless, despite robust activity in viral RNA synthesis, we were unable to rescue any of the corresponding viruses. However, as for some of our directly fused NP constructs, these L constructs directly fused at their N-terminus to fluorescent reporters will still provide valuable tools for imaging approaches, particularly those focused on understanding the formation and function of arenavirus replication complexes. In contrast, C-terminal fusion of the reporters resulted in an L protein that was strongly impaired or, in most cases, completely nonfunctional. Further, these C-terminal directly fused L proteins seem to show some evidence of aggregation within the cytoplasm, particularly for the GFP-fused construct, suggesting that problems with folding or oligomerization may contribute to their lack of function. This is consistent with previously published observations that adding tags to the C-terminus of LASV-L also impairs its activity by modifying the protein conformation [81]. Given these difficulties in generating terminal fusions to L, we finally considered a strategy that was initially described for Measles virus [46], but has also successfully been used for other NSVs, such as Vesicular stomatitis virus [47] and in our own work on Ebola virus [82], in which reporter genes can be inserted into a flexible hinge region within the polymerase. Indeed, for LASV L, a similar domain structure, composed of discrete domains connected by flexible linker regions, is also experimentally supported [81]. As expected, based on previous studies with these other NSVs, internally fused TCRV L proteins showed activity comparable with wild-type in a minigenome assay, and the corresponding viruses could be readily rescued. However, despite only modest attenuation of virus growth, reporter expression was surprisingly poor in comparison to results from these other systems [46,47,82]. While this might be due to comparatively lower expression levels of the arenavirus polymerase, for instance as a result of their fundamentally different transcription strategies, studies on LASV L have also suggested that tags placed in this region may themselves reduce expression of L [81]. Nonetheless, reporter expression was still clearly detectable following infection with a recombinant TCRV expressing an internal L fusion with nLuc, indicating that this strategy is indeed viable, but that its practical utility appears to be primarily determined by the sensitivity that can be achieved during reporter detection. In this context, the continuous efforts being made to generate increasingly bright fluorescent protein derivatives (e.g., mGreenLantern, SuperTag-RFP) [83,84] will likely support the future success of recombinant fluorescent arenavirus generation based on this approach.

Overall, several of the strategies evaluated here were found to be viable, and in particular, led to the creation of direct fusion proteins that retain key functions (i.e., C-terminal fusions with NP, C-terminal fusions with Z, and N-terminal or internal fusions with L) and in some cases could even be used to generate viable recombinant TCRVs (i.e., using C-terminal fusions with Z, and internal fusions with L). These resources are themselves of great value for future research, particularly due to their suitability for use in imaging applications that have the potential to significantly advance our understanding of spatiotemporal dynamics of viral protein localization and trafficking. Further, the systematic approach used in our study has revealed a number of insights regarding the impact of the location and size of the reporter genes, reporter sensitivity, as well as the suitability of different 2A self-cleavage sites for recombinant arenavirus construction that will inform future studies and allow the construction of reporter-expressing arenaviruses more suitable for specific applications.

## Figures and Tables

**Table 1:** Summary of strategies to generate TCRV reporter proteins and viruses.

Protein	Fusion Site	Protein Function		Virus Viability		
		Direct Fusion	T2A Fusion	Direct Fusion	T2A Fusion	Separate ORF
Tri segmented	∆NP					+
	∆GPC					+
NP	N-terminal	−/+	+	−	+	
	C-terminal	+ ^a^	+	−	−	
GPC	C-terminal	−	−/+	−	−	
Z	C-terminal	+	+	+	+	
L	N-terminal	+ ^b^	+	−	−	
	C-terminal	−	−	n.d.	n.d.	
	Internal	+	+	+ ^c^		

+ yes, −/+ partial activity, − no, n.d. not done, ^a^ except for GFP, ^b^ except for nLuc, ^c^ reporter expression detectable for nLuc only.

## Data Availability

All data are included in this published article (and its supplementary files).

## Supplementary Materials

The following supporting information can be downloaded at: www.mdpi.com/article/10.3390/v14071563/s1, Figure S1: Arenavirus polymerase (L) sequence alignment.

## References

[1] Hallam S.J., Koma T., Maruyama J., Paessler S. (2018). Review of Mammarenavirus Biology and Replication. Front. Microbiol..

[2] Radoshitzky S.R., Abraham J., Spiropoulou C.F., Kuhn J.H., Nguyen D., Li W., Nagel J., Schmidt P.J., Nunberg J.H., Andrews N.C. (2007). Transferrin receptor 1 is a cellular receptor for New World haemorrhagic fever arenaviruses. Nature.

[3] Abraham J., Kwong J.A., Albarino C.G., Lu J.G., Radoshitzky S.R., Salazar-Bravo J., Farzan M., Spiropoulou C.F., Choe H. (2009). Host-species transferrin receptor 1 orthologs are cellular receptors for nonpathogenic new world clade B arenaviruses. PLoS Pathog..

[4] Castilla V., Mersich S.E., Candurra N.A., Damonte E.B. (1994). The entry of Junin virus into Vero cells. Arch. Virol..

[5] Martinez M.G., Cordo S.M., Candurra N.A. (2007). Characterization of Junin arenavirus cell entry. J. Gen. Virol..

[6] Kang H.L., Cong J.Y., Wang C.L., Ji W.X., Xin Y.H., Qian Y., Li X.M., Chen Y.T., Rao Z.H. (2021). Structural basis for recognition and regulation of arenavirus polymerase L by Z protein. Nat. Commun..

[7] Wolff S., Ebihara H., Groseth A. (2013). Arenavirus budding: A common pathway with mechanistic differences. Viruses.

[8] Rojek J.M., Lee A.M., Nguyen N., Spiropoulou C.F., Kunz S. (2008). Site 1 protease is required for proteolytic processing of the glycoproteins of the South American hemorrhagic fever viruses Junin, Machupo, and Guanarito. J. Virol..

[9] York J., Nunberg J.H. (2006). Role of the stable signal peptide of Junin arenavirus envelope glycoprotein in pH-dependent membrane fusion. J. Virol..

[10] Enria D.A., Briggiler A.M., Feuillade M.R. (1998). An overview of the epidemiological, ecological and preventive hallmarks of Argentine haemorrhagic fever (Junin virus). Bull. L’institut Pasteur.

[11] Patterson M., Grant A., Paessler S. (2014). Epidemiology and pathogenesis of Bolivian hemorrhagic fever. Curr. Opin. Virol..

[12] Rodriguez-Morales A.J., Bonilla-Aldana D.K., Risquez A., Paniz-Mondolfi A., Suarez J.A. (2021). Should we be concerned about Venezuelan hemorrhagic fever?-A reflection on its current situation in Venezuela and potential impact in Latin America amid the migration crisis. New Microbes New Infect..

[13] Enria D.A., Briggiler A.M., Sanchez Z. (2008). Treatment of Argentine hemorrhagic fever. Antivir. Res..

[14] Sociedad Argentina de Vacunología y Epidemiologia Sociedad Argentina de Virología.; Subcomisión Vacunología de la Asociación Argentina de Microbiología. https://save.org.ar/wp-content/uploads/2019/07/Documento-Posicion-Fiebre-Hemorragica-Argentina-.pdf.

[15] Fénéant L., Bodmer B., Mettenleiter T.C., Groseth A., Hoenen T. (2020). Current Therapies for Biosafety Level 4 Pathogens. New Developments in Antiviral Drugs.

[16] Dunham E.C., Leske A., Shifflett K., Watt A., Feldmann H., Hoenen T., Groseth A. (2018). Lifecycle modelling systems support inosine monophosphate dehydrogenase (IMPDH) as a pro-viral factor and antiviral target for New World arenaviruses. Antivir. Res..

[17] Martin S., Chiramel A.I., Schmidt M.L., Chen Y.C., Whitt N., Watt A., Dunham E.C., Shifflett K., Traeger S., Leske A. (2018). A genome-wide siRNA screen identifies a druggable host pathway essential for the Ebola virus life cycle. Genome Med..

[18] Lee N., Shum D., Konig A., Kim H., Heo J., Min S., Lee J., Ko Y., Choi I., Lee H. (2018). High-throughput drug screening using the Ebola virus transcription- and replication-competent virus-like particle system. Antivir. Res..

[19] Lavanya M., Cuevas C.D., Thomas M., Cherry S., Ross S.R. (2013). siRNA screen for genes that affect Junin virus entry uncovers voltage-gated calcium channels as a therapeutic target. Sci. Transl. Med..

[20] Edwards M.R., Pietzsch C., Vausselin T., Shaw M.L., Bukreyev A., Basler C.F. (2015). High-Throughput Minigenome System for Identifying Small-Molecule Inhibitors of Ebola Virus Replication. ACS Infect. Dis..

[21] Sanchez-Velazquez R., de Lorenzo G., Tandavanitj R., Setthapramote C., Bredenbeek P.J., Bozzacco L., MacDonald M.R., Clark J.J., Rice C.M., Patel A.H. (2020). Generation of a reporter yellow fever virus for high throughput antiviral assays. Antivir. Res..

[22] Zhang Z.R., Zhang H.Q., Li X.D., Deng C.L., Wang Z., Li J.Q., Li N., Zhang Q.Y., Zhang H.L., Zhang B. (2020). Generation and characterization of Japanese encephalitis virus expressing GFP reporter gene for high throughput drug screening. Antivir. Res..

[23] Zou G., Xu H.Y., Qing M., Wang Q.Y., Shi P.Y. (2011). Development and characterization of a stable luciferase dengue virus for high-throughput screening. Antivir. Res..

[24] Reuther P., Gopfert K., Dudek A.H., Heiner M., Herold S., Schwemmle M. (2015). Generation of a variety of stable Influenza A reporter viruses by genetic engineering of the NS gene segment. Sci. Rep..

[25] Li L.H., Kaptein S.J.F., Schmid M.A., Zmurko J., Leyssen P., Neyts J., Dallmeier K. (2020). A dengue type 2 reporter virus assay amenable to high-throughput screening. Antivir. Res..

[26] Dorjsuren D., Eastman R.T., Song M.J., Yasgar A., Chen Y., Bharti K., Zakharov A.V., Jadhav A., Ferrer M., Shi P.Y. (2022). A platform of assays for the discovery of anti-Zika small-molecules with activity in a 3D-bioprinted outer-blood-retina model. PLoS ONE.

[27] Chiem K., Morales Vasquez D., Park J.G., Platt R.N., Anderson T., Walter M.R., Kobie J.J., Ye C., Martinez-Sobrido L. (2021). Generation and Characterization of recombinant SARS-CoV-2 expressing reporter genes. J. Virol..

[28] Johansen L.M., Brannan J.M., Delos S.E., Shoemaker C.J., Stossel A., Lear C., Hoffstrom B.G., Dewald L.E., Schornberg K.L., Scully C. (2013). FDA-approved selective estrogen receptor modulators inhibit Ebola virus infection. Sci. Transl. Med..

[29] Bennett R.P., Finch C.L., Postnikova E.N., Stewart R.A., Cai Y., Yu S., Liang J., Dyall J., Salter J.D., Smith H.C. (2021). A Novel Ebola Virus VP40 Matrix Protein-Based Screening for Identification of Novel Candidate Medical Countermeasures. Viruses.

[30] Liu Y., Lee M.S., Olson M.A., Harty R.N. (2011). Bimolecular Complementation to Visualize Filovirus VP40-Host Complexes in Live Mammalian Cells: Toward the Identification of Budding Inhibitors. Adv. Virol..

[31] Konig R., Stertz S., Zhou Y., Inoue A., Hoffmann H.H., Bhattacharyya S., Alamares J.G., Tscherne D.M., Ortigoza M.B., Liang Y. (2010). Human host factors required for influenza virus replication. Nature.

[32] Panda D., Rose P.P., Hanna S.L., Gold B., Hopkins K.C., Lyde R.B., Marks M.S., Cherry S. (2013). Genome-wide RNAi screen identifies SEC61A and VCP as conserved regulators of Sindbis virus entry. Cell Rep..

[33] Ramage H.R., Kumar G.R., Verschueren E., Johnson J.R., Von Dollen J., Johnson T., Newton B., Shah P., Horner J., Krogan N.J. (2015). A combined proteomics/genomics approach links hepatitis C virus infection with nonsense-mediated mRNA decay. Mol. Cell.

[34] McCormick D., Lin Y.T., Grey F. (2018). Identification of Host Factors Involved in Human Cytomegalovirus Replication, Assembly, and Egress Using a Two-Step Small Interfering RNA Screen. mBio.

[35] Ngo N., Henthorn K.S., Cisneros M.I., Cubitt B., Iwasaki M., de la Torre J.C., Lama J. (2015). Identification and Mechanism of Action of a Novel Small-Molecule Inhibitor of Arenavirus Multiplication. J. Virol..

[36] Welch S.R., Spengler J.R., Genzer S.C., Chatterjee P., Flint M., Bergeron E., Montgomery J.M., Nichol S.T., Albarino C.G., Spiropoulou C.F. (2021). Screening and Identification of Lujo Virus Inhibitors Using a Recombinant Reporter Virus Platform. Viruses.

[37] Cai Y., Iwasaki M., Beitzel B.F., Yu S., Postnikova E.N., Cubitt B., DeWald L.E., Radoshitzky S.R., Bollinger L., Jahrling P.B. (2018). Recombinant Lassa Virus Expressing Green Fluorescent Protein as a Tool for High-Throughput Drug Screens and Neutralizing Antibody Assays. Viruses.

[38] Emonet S.F., Garidou L., McGavern D.B., de la Torre J.C. (2009). Generation of recombinant lymphocytic choriomeningitis viruses with trisegmented genomes stably expressing two additional genes of interest. Proc. Natl. Acad. Sci. USA.

[39] Emonet S.F., Seregin A.V., Yun N.E., Poussard A.L., Walker A.G., de la Torre J.C., Paessler S. (2011). Rescue from cloned cDNAs and in vivo characterization of recombinant pathogenic Romero and live-attenuated Candid #1 strains of Junin virus, the causative agent of Argentine hemorrhagic fever disease. J. Virol..

[40] Popkin D.L., Teijaro J.R., Lee A.M., Lewicki H., Emonet S., de la Torre J.C., Oldstone M. (2011). Expanded potential for recombinant trisegmented lymphocytic choriomeningitis viruses: Protein production, antibody production, and in vivo assessment of biological function of genes of interest. J. Virol..

[41] Ye C.J., de la Torre J.C., Martinez-Sobrido L. (2020). Development of Reverse Genetics for the Prototype New World Mammarenavirus Tacaribe Virus. J. Virol..

[42] Ortiz-Riano E., Cheng B.Y.H., Carlos de la Torre J., Martinez-Sobrido L. (2013). Arenavirus reverse genetics for vaccine development. J. Gen. Virol..

[43] Dhanwani R., Zhou Y., Huang Q., Verma V., Dileepan M., Ly H., Liang Y. (2015). A Novel Live Pichinde Virus-Based Vaccine Vector Induces Enhanced Humoral and Cellular Immunity after a Booster Dose. J. Virol..

[44] Wan W., Zhu S., Li S., Shang W., Zhang R., Li H., Liu W., Xiao G., Peng K., Zhang L. (2021). High-Throughput Screening of an FDA-Approved Drug Library Identifies Inhibitors against Arenaviruses and SARS-CoV-2. ACS Infect. Dis..

[45] Groseth A., Wolff S., Strecker T., Hoenen T., Becker S. (2010). Efficient budding of the tacaribe virus matrix protein z requires the nucleoprotein. J. Virol..

[46] Duprex W.P., Collins F.M., Rima B.K. (2002). Modulating the function of the measles virus RNA-dependent RNA polymerase by insertion of green fluorescent protein into the open reading frame. J. Virol..

[47] Ruedas J.B., Perrault J. (2009). Insertion of Enhanced Green Fluorescent Protein in a Hinge Region of Vesicular Stomatitis Virus L Polymerase Protein Creates a Temperature-Sensitive Virus That Displays No Virion-Associated Polymerase Activity In Vitro. J. Virol..

[48] Fix J., Galloux M., Blondot M.L., Eleouet J.F. (2011). The insertion of fluorescent proteins in a variable region of respiratory syncytial virus L polymerase results in fluorescent and functional enzymes but with reduced activities. Open Virol. J..

[49] Groseth A., Feldmann H., Theriault S., Mehmetoglu G., Flick R. (2005). RNA polymerase I-driven minigenome system for Ebola viruses. J. Virol..

[50] Wolff S., Groseth A., Meyer B., Jackson D., Strecker T., Kaufmann A., Becker S. (2016). The New World arenavirus Tacaribe virus induces caspase-dependent apoptosis in infected cells. J. Gen. Virol..

[51] Holzerland J., Leske A., Feneant L., Garcin D., Kolakofsky D., Groseth A. (2020). Complete genome sequence of Tacaribe virus. Arch. Virol..

[52] Albarino C.G., Bergeron E., Erickson B.R., Khristova M.L., Rollin P.E., Nichol S.T. (2009). Efficient reverse genetics generation of infectious junin viruses differing in glycoprotein processing. J. Virol..

[53] Enterlein S., Volchkov V., Weik M., Kolesnikova L., Volchkova V., Klenk H.D., Muhlberger E. (2006). Rescue of recombinant Marburg virus from cDNA is dependent on nucleocapsid protein VP30. J. Virol..

[54] Neumann G., Feldmann H., Watanabe S., Lukashevich I., Kawaoka Y. (2002). Reverse genetics demonstrates that proteolytic processing of the Ebola virus glycoprotein is not essential for replication in cell culture. J. Virol..

[55] Kato A., Sakai Y., Shioda T., Kondo T., Nakanishi M., Nagai Y. (1996). Initiation of Sendai virus multiplication from transfected cDNA or RNA with negative or positive sense. Genes Cells.

[56] Durbin A.P., Hall S.L., Siew J.W., Whitehead S.S., Collins P.L., Murphy B.R. (1997). Recovery of infectious human parainfluenza virus type 3 from cDNA. Virology.

[57] Brown J.D., Ryan M.D., Atkins J.F., Gesteland R.F. (2010). Ribosome “Skipping”: “Stop-Carry On” or “StopGo” Translation. Recoding: Expansion of Decoding Rules Enriches Gene Expression.

[58] Baird N.L., York J., Nunberg J.H. (2012). Arenavirus Infection Induces Discrete Cytosolic Structures for RNA Replication. J. Virol..

[59] Perez M., Greenwald D.L., de La Torre J.C. (2004). Myristoylation of the RING finger Z protein is essential for arenavirus budding. J. Virol..

[60] Shi X., van Mierlo J.T., French A., Elliott R.M. (2010). Visualizing the replication cycle of bunyamwera orthobunyavirus expressing fluorescent protein-tagged Gc glycoprotein. J. Virol..

[61] Hotard A.L., Shaikh F.Y., Lee S., Yan D., Teng M.N., Plemper R.K., Crowe J.E., Moore M.L. (2012). A stabilized respiratory syncytial virus reverse genetics system amenable to recombination-mediated mutagenesis. Virology.

[62] Falchieri M., Lupini C., Cecchinato M., Catelli E., Kontolaimou M., Naylor C.J. (2013). Avian metapneumoviruses expressing Infectious Bronchitis virus genes are stable and induce protection. Vaccine.

[63] Tokusumi T., Iida A., Hirata T., Kato A., Nagai Y., Hasegawa M. (2002). Recombinant Sendai viruses expressing different levels of a foreign reporter gene. Virus Res..

[64] Cheng B.Y., Ortiz-Riano E., de la Torre J.C., Martinez-Sobrido L. (2015). Arenavirus Genome Rearrangement for the Development of Live Attenuated Vaccines. J. Virol..

[65] Kim M.S., Park J.S., Kim K.H. (2013). Optimal place of a foreign gene in the genome of viral haemorrhagic septicaemia virus (VHSV) for development of VHSV-based viral-vectored vaccines. J. Appl. Microbiol..

[66] Wertz G.W., Moudy R., Ball L.A. (2002). Adding genes to the RNA genome of vesicular stomatitis virus: Positional effects on stability of expression. J. Virol..

[67] Goto H., Muramoto Y., Noda T., Kawaoka Y. (2013). The Genome-Packaging Signal of the Influenza A Virus Genome Comprises a Genome Incorporation Signal and a Genome-Bundling Signal. J. Virol..

[68] Pinschewer D.D., Perez M., de la Torre J.C. (2005). Dual role of the lymphocytic choriomeningitis virus intergenic region in transcription termination and virus propagation. J. Virol..

[69] Dhanwani R., Ly H., Liang Y. (2017). Recombinant Tri-Segmented Pichinde Virus as a Novel Live Viral Vaccine Platform. Methods Mol. Biol..

[70] Donnelly M.L.L., Hughes L.E., Luke G., Mendoza H., Ten Dam E., Gani D., Ryan M.D. (2001). The ‘cleavage’ activities of foot-and-mouth disease virus 2A site-directed mutants and naturally occurring ‘2A-like’ sequences. J. Gen. Virol..

[71] Kim J.H., Lee S.R., Li L.H., Park H.J., Park J.H., Lee K.Y., Kim M.K., Shin B.A., Choi S.Y. (2011). High Cleavage Efficiency of a 2A Peptide Derived from Porcine Teschovirus-1 in Human Cell Lines, Zebrafish and Mice. PLoS ONE.

[72] D’Antuono A., Loureiro M.E., Foscaldi S., Marino-Buslje C., Lopez N. (2014). Differential contributions of tacaribe arenavirus nucleoprotein N-terminal and C-terminal residues to nucleocapsid functional activity. J. Virol..

[73] Casabona J.C., Macleod J.M.L., Loureiro M.E., Gomez G.A., Lopez N. (2009). The RING Domain and the L79 Residue of Z Protein Are Involved in both the Rescue of Nucleocapsids and the Incorporation of Glycoproteins into Infectious Chimeric Arenavirus-Like Particles. J. Virol..

[74] Levingston Macleod J.M., D’Antuono A., Loureiro M.E., Casabona J.C., Gomez G.A., Lopez N. (2011). Identification of two functional domains within the arenavirus nucleoprotein. J. Virol..

[75] Takamatsu Y., Kajikawa J., Muramoto Y., Nakano M., Noda T. (2019). Microtubule-dependent transport of arenavirus matrix protein demonstrated using live-cell imaging microscopy. Microscopy.

[76] Markosyan R.M., Marin M., Zhang Y., Cohen F.S., Melikyan G.B. (2021). The late endosome-resident lipid bis(monoacylglycero)phosphate is a cofactor for Lassa virus fusion. PLoS Pathog..

[77] Hulseberg C.E., Feneant L., Szymanska K.M., White J.M. (2018). Lamp1 Increases the Efficiency of Lassa Virus Infection by Promoting Fusion in Less Acidic Endosomal Compartments. mBio.

[78] Raaben M., Jae L.T., Herbert A.S., Kuehne A.I., Stubbs S.H., Chou Y.Y., Blomen V.A., Kirchhausen T., Dye J.M., Brummelkamp T.R. (2017). NRP2 and CD63 Are Host Factors for Lujo Virus Cell Entry. Cell Host Microbe.

[79] Watt A., Moukambi F., Banadyga L., Groseth A., Callison J., Herwig A., Ebihara H., Feldmann H., Hoenen T. (2014). A novel life cycle modeling system for Ebola virus shows a genome length-dependent role of VP24 in virus infectivity. J. Virol..

[80] Urata S., Yasuda J., de la Torre J.C. (2009). The z protein of the new world arenavirus tacaribe virus has bona fide budding activity that does not depend on known late domain motifs. J. Virol..

[81] Vogel D., Rosenthal M., Gogrefe N., Reindl S., Gunther S. (2019). Biochemical characterization of the Lassa virus L protein. J. Biol. Chem..

[82] Hoenen T., Shabman R.S., Groseth A., Herwig A., Weber M., Schudt G., Dolnik O., Basler C.F., Becker S., Feldmann H. (2012). Inclusion Bodies Are a Site of Ebolavirus Replication. J. Virol..

[83] Campbell B.C., Nabel E.M., Murdock M.H., Lao-Peregrin C., Tsoulfas P., Blackmore M.G., Lee F.S., Liston C., Morishita H., Petsko G.A. (2020). mGreenLantern: A bright monomeric fluorescent protein with rapid expression and cell filling properties for neuronal imaging. Proc. Natl. Acad. Sci. USA.

[84] Mo G.C.H., Posner C., Rodriguez E.A., Sun T., Zhang J. (2020). A rationally enhanced red fluorescent protein expands the utility of FRET biosensors. Nat. Commun..

