# Supplementary figures and images for: Generation of Reporter-Expressing New World Arenaviruses: A Systematic Comparison
Source: Viruses. 2022 Jul 18;14(7):1563. doi: 10.3390/v14071563 (PMC9317149; doi:10.3390/v14071563)

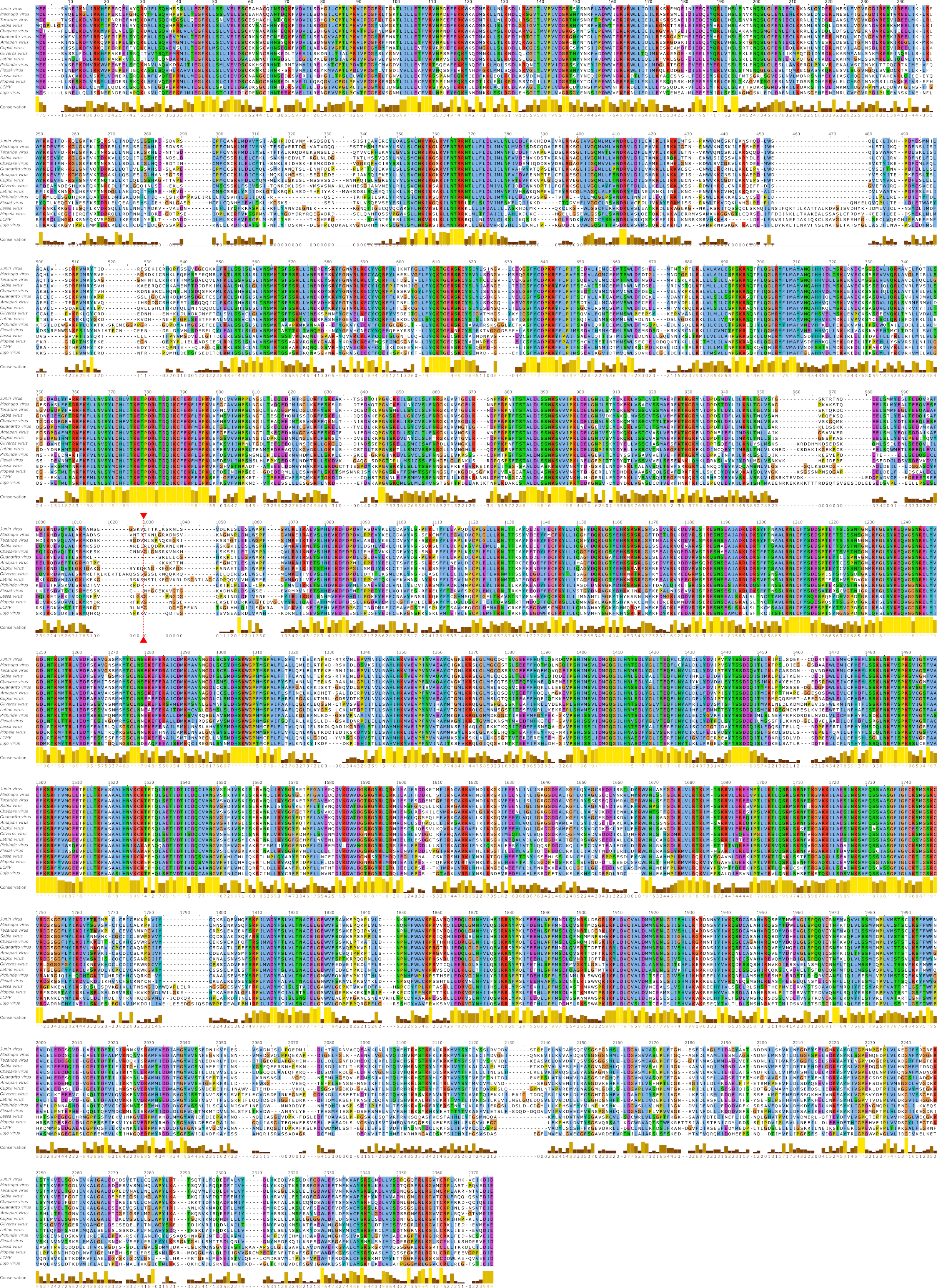

Supplement: Supplementary file 1 [file viruses-14-01563-s001.zip › viruses-1788749-supplementary.tif]
